# Controlled coupling of an ultrapotent auristatin warhead to cetuximab yields a next-generation antibody-drug conjugate for EGFR-targeted therapy of KRAS mutant pancreatic cancer

**DOI:** 10.1038/s41416-020-01046-6

**Published:** 2020-09-11

**Authors:** Michelle K. Greene, Ting Chen, Eifion Robinson, Ninfa L. Straubinger, Charlene Minx, Darren K. W. Chan, Jun Wang, James F. Burrows, Sandra Van Schaeybroeck, James R. Baker, Stephen Caddick, Daniel B. Longley, Donald E. Mager, Robert M. Straubinger, Vijay Chudasama, Christopher J. Scott

**Affiliations:** 1 The Patrick G Johnston Centre for Cancer Research, School of Medicine, Dentistry and Biomedical Sciences, Queen’s University Belfast, Belfast, UK; 2 Department of Pharmaceutical Sciences, University at Buffalo, Buffalo, NY USA; 3 Department of Chemistry, University College London, London, UK; 4 School of Pharmacy, Queen’s University Belfast, Belfast, UK; 5 Department of Pharmacology & Cancer Therapeutics, Roswell Park Cancer Institute, Buffalo, NY USA

**Keywords:** Targeted therapies, Chemical modification

## Abstract

**Background:**

Antibody-drug conjugate (ADC) construction poses numerous challenges that limit clinical progress. In particular, common bioconjugation methods afford minimal control over the site of drug coupling to antibodies. Here, such difficulties are overcome through re-bridging of the inter-chain disulfides of cetuximab (CTX) with auristatin-bearing pyridazinediones, to yield a highly refined anti-epidermal growth factor receptor (EGFR) ADC.

**Methods:**

In vitro and in vivo assessment of ADC activity was performed in KRAS mutant pancreatic cancer (PaCa) models with known resistance to CTX therapy. Computational modelling was employed for quantitative prediction of tumour response to various ADC dosing regimens.

**Results:**

Site-selective coupling of an auristatin to CTX yielded an ADC with an average drug:antibody ratio (DAR) of 3.9, which elicited concentration- and EGFR-dependent cytotoxicity at sub-nanomolar potency in vitro. In human xenografts, the ADC inhibited tumour growth and prolonged survival, with no overt signs of toxicity. Key insights into factors governing ADC efficacy were obtained through a robust mathematical framework, including target-mediated dispositional effects relating to antigen density on tumour cells.

**Conclusions:**

Together, our findings offer renewed hope for CTX in PaCa therapy, demonstrating that it may be reformatted as a next-generation ADC and combined with a predictive modelling tool to guide successful translation.

## Background

Pancreatic cancer (PaCa) poses a significant clinical oncology challenge because of frequent high levels of resistance to multiple different therapeutic interventions. Recent statistics indicate that PaCa is the 4th leading cause of cancer-related death, with 55,440 new diagnoses and 44,330 fatalities estimated for the USA in 2018.^1^ Due to the largely asymptomatic nature of PaCa and the lack of specific biomarkers to aid detection, most cases remain undiagnosed until advanced stages, when patients are no longer eligible for curative resection. Frontline treatment options for these patients are limited and often involve toxic drug combinations that confer modest clinical benefit at most, extending survival by a matter of weeks. The prognosis for PaCa patients is therefore remarkably poor, with a 5-year relative survival rate of 8% that has scarcely improved over several decades, clearly highlighting the need for novel therapeutic approaches.^1^

Antibody-drug conjugates (ADCs), which typically comprise a full IgG molecule linked to cytotoxic payloads, are one of the fastest-growing classes of biotherapeutics, and have the potential to revolutionise PaCa therapy.^2–4^ These agents exploit the targeting ability of antibodies to deliver a highly potent payload selectively to antigen-expressing cells. This targeting can greatly enhance the therapeutic index of attached cargoes that are otherwise too toxic for use as single agents. Although ADCs were first investigated in humans in the 1980s, it is only within the last decade that they have excelled in the clinic, leading to the marketing approval of several conjugates. These are mainly indicated for either breast or haematological malignancies, with no ADCs yet approved for PaCa therapy.

Despite the recent success of ADCs, efforts aimed at refining their synthesis remain a key priority. A notable design constraint of many ADCs is the choice of bioconjugation chemistry for coupling the drug-linker entity to the antibody. Traditionally, this has been achieved using amine-reactive linkers that mediate random drug conjugation to lysine side-chains *via* amide bond formation. However, the high abundance of lysine residues throughout antibodies affords minimal control over the site of conjugation, leading to heterogeneous mixtures of several ADC species that may differ significantly in terms of stability, pharmacokinetics (PK), drug:antibody ratio (DAR) and potency.^5^ Alternatively, cysteine residues have also been commonly targeted for bioconjugation purposes, by reacting maleimide-containing linkers with sulfhydryls liberated from the reduction of inter-chain disulfide bonds. This approach also presents challenges, in that the resultant thiosuccinimide adducts are susceptible to retro-Michael deconjugation in the circulation, leading to premature drug dissociation and systemic toxicity.^6,7^ In addition, this approach generates heterogeneous mixtures when targeting typical DARs of 2–4 as the four inter-chain disulfide bonds cannot be reduced selectively.

Given these difficulties, much attention is currently focused on the development of superior bioconjugation approaches that allow for the controlled and site-specific coupling of cytotoxic cargoes to antibodies.^8–14^ Previously, we have shown that inter-chain disulfides within the human epidermal growth factor receptor 2 (HER2)-targeted antibody trastuzumab may be selectively re-bridged with dibromopyridazinedione (diBrPD)-based linkers bearing monomethylauristatin E (MMAE) payloads, to yield highly uniform and serum-stable ADCs with therapeutic activity in breast cancer models.^15^ Here, we provide the first demonstration that ADC synthesis using this diBrPD-MMAE drug-linker may be successfully translated to both another antibody platform and tumour indication, allowing us to arm epidermal growth factor receptor (EGFR)-targeted cetuximab (CTX) with an ultrapotent MMAE warhead for application in PaCa (hereafter referred to as CTX-MMAE). We show that CTX-MMAE is well-tolerated and specifically targets EGFR to elicit dose-dependent therapeutic effects in two distinct models of PaCa that harbour KRAS mutations, which render them refractory to standard EGFR-targeted therapies. Moreover, through the development of a population kinetic-pharmacodynamic (K-PD) model that quantitatively describes the dose-response relationship of CTX-MMAE in these two in vivo models of PaCa, we have generated a valuable predictive tool that provides mechanistic insights into key determinants of ADC efficacy and can be used to inform the future optimisation of the CTX-MMAE dosing regimen as it progresses through subsequent development.

## Methods

### Bioconjugation of MMAE to CTX

A solution of CTX (3000 µL of a 40 μM solution in borate buffer (BBS) pH 8, 0.12 µmol (1 eq)) was split into equal volumes (500 µL, 0.02 µmol) in six Eppendorf tubes, and to each tube was added a solution of TCEP (6 × 12 μL of a 10 mM solution in BBS pH 8, 6 × 0.12 µmol (6 eq)). The reaction mixtures were incubated at 37 °C/450 rpm for 90 min. The reaction mixtures were then cooled to 4 °C using an ice bath and to each vial was added a cooled solution of diBrPD-PEG12-valine-citrulline-*p*-aminobenzyloxycarbonyl (PABC)-MMAE (6 × 80 μL of a 10 mM solution in DMF, 6 × 0.80 µmol (40 eq)). Synthetic procedures for the diBrPD-PEG12-valine-citrulline-PABC-MMAE re-bridging reagent and the diBrPD(Me)-acid precursor were as previously described.^12,15^ The reaction mixtures were left to stand at 4 °C for 18 h, then buffer swapped repeatedly (6×) into PBS pH 7.4, making up to a final volume of 2500 µL, of which 35 µL was diluted 1/2 and 5 μL was diluted 1/20 for UV-VIS analysis. An 83% yield of CTX-MMAE was obtained.

### Sodium dodecyl sulphate polyacrylamide gel electrophoresis (SDS-PAGE)

Non-reducing SDS-PAGE (10%) was performed following standard lab procedures. A 4% stacking gel was used and a broad-range molecular weight marker (3–198 kDa, Prestained SeeBlue Plus 2 protein standard, ThermoScientific) was co-run to estimate protein weights. Samples (10 μL at 7 μM) were mixed with loading buffer (2 μL, composition for 5x SDS: 1 g SDS, 3 mL glycerol, 6 mL 0.5 M Tris buffer pH 6.8, 2 mg bromophenol blue in 10 mL), heated at 75 °C for 5 min, and centrifuged at 16,000 rpm for 5 min. Samples were subsequently loaded into the wells in a volume of 5 μL. Gels were stained using InstantBlue protein stain (Expedeon).

### Hydrophobic interaction chromatography (HIC)

A sample of CTX-MMAE (~35 μM) was diluted two times with water and injected (6–12 µL) onto a TSK-Gel Butyl-NPR 4.6 mm × 35 mm, 2.5 µm particle size column from Tosoh Bioscience, connected to an Agilent 1100 HPLC equipped with a diode array for UV-VIS detection. Samples were run with a step gradient from 100% buffer A (1.5 M ammonium sulfate, 25 mM sodium phosphate, pH 7) to 45% buffer B (25 mM sodium phosphate, 25% isopropanol (v/v), pH 7) over 52 min at a flow rate of 0.6 mL/min. The temperature was maintained at 20 °C for the duration of the run. Detection was by UV-VIS absorbance at 280 nm.

### General cell culture

MIA PaCa-2 and PANC-1 human PaCa cell lines were obtained from the American Type Culture Collection (ATCC), USA, and cultured in complete DMEM (DMEM supplemented with 1 mM sodium pyruvate, 50 units/mL penicillin, 50 µg/mL streptomycin and 10% (v/v) foetal bovine serum (FBS)). Both cell lines were maintained in 5% CO_2_ at 37 °C in a humidified incubator.

### Immunoblotting

MIA PaCa-2 and PANC-1 cells were lysed in RIPA buffer supplemented with cOmplete™ mini protease inhibitor cocktail (Roche). Following incubation for 30 min on ice, lysates were centrifuged at 20,000×*g* for 10 min at 4 °C and the supernatant was collected for quantification of protein content using the BCA protein assay kit (Thermo Scientific). Samples were denatured for 10 min at 95 °C, separated by SDS-PAGE and transferred onto a PVDF membrane (Millipore). After immersion in tris-buffered saline containing 0.1% (v/v) Tween 20 and 5% (w/v) bovine serum albumin (blocking solution) for 1 h at room temperature, the membrane was probed with rabbit anti-EGFR (Cell Signaling Technology; 1:1000 in blocking solution) or rat anti-tubulin (Abcam; 1:1000 in blocking solution) primary antibodies overnight at 4 °C. Following incubation with horseradish peroxidase (HRP)-conjugated goat anti-rabbit (Cell Signaling Technology; 1:10,000 in blocking solution) or rabbit anti-rat (Abcam; 1:10,000 in blocking solution) secondary antibodies for 1 h at room temperature, the membrane was overlaid with Immobilon® Forte Western HRP substrate (Millipore) and protein expression was imaged using the ChemiDoc XRS+ system (Bio-Rad).

### Thiazolyl blue tetrazolium bromide (MTT) cell viability assay

MIA PaCa-2 and PANC-1 cells were seeded at 1000 and 1500 per well, respectively, in a 96-well plate and left to adhere overnight. For concentration-response studies, cells were treated with a 5-fold dilution series of CTX-MMAE or CTX ranging from 0.000256 to 500 nM for 96 h. For EGFR targeting specificity studies, cells were treated with 5 nM CTX-MMAE and a 5-fold dilution series of competing CTX ranging from 0.1 to 343 nM for 96 h. After treatment, MTT was added to the culture media at a final concentration of 0.5 mg/mL for 3 h and formazan crystals were then dissolved in DMSO, followed by measurement of absorbance at 570 nm. Results are presented as percentage viability relative to PBS-treated cells.

### Clonogenic assay

MIA PaCa-2 and PANC-1 cells were seeded at 250 and 500 per well, respectively, in a 6-well plate and left to adhere overnight. For concentration-response studies, cells were treated with a 10-fold dilution series of CTX-MMAE or CTX ranging from 0.00005 to 50 nM. For EGFR targeting specificity studies, cells were treated with 0.5 nM CTX-MMAE and a 5-fold dilution series of competing CTX ranging from 0.0224 to 70 nM. Cells were then incubated for 8–14 days with minimal disturbance to allow colony formation. At study endpoint, cells were washed in PBS and stained in 0.4% (w/v) crystal violet solution.

### EGFR depletion

An EGFR-targeted and a negative control siRNA (Qiagen) were transfected into PANC-1 cells using HiPerFect reagent (Qiagen), in accordance with the manufacturer’s instructions. Briefly, siRNA (50 µL of a 1 µM solution in RNase-free water) was spotted onto the centre of a 60 mm dish and then overlaid with a mixture of HiPerFect transfection reagent (15 µL) and Opti-MEM reduced serum medium (1 mL; Gibco). Following incubation for 30 min at 37 °C to allow formation of transfection complexes, a suspension of PANC-1 cells (5 × 10^5^) in complete DMEM (4 mL) was added to the dish. After 24 h, the cells were detached from plasticware by incubation in 0.1% (w/v) EDTA in PBS for 10 min at 37 °C and then re-seeded at 2 × 10^5^ per 60 mm dish. Cells were left for a further 48 h prior to confirmation of EGFR knockdown by flow cytometry and subsequent exposure to CTX-MMAE.

### Flow cytometry

At 72 h following transfection, PANC-1 cells were washed (2×) in PBS and detached from plasticware by incubation in 0.1% (w/v) EDTA in PBS for 10 min at 37 °C. Cells were centrifuged at 200×*g* for 5 min at 4 °C, resuspended in FACS buffer (5% (v/v) FBS in PBS) and incubated with FITC-labelled anti-human EGFR (5 µg/mL; Santa Cruz Biotechnology) or FITC-labelled anti-mouse IgG2a isotype control (5 µg/mL; Santa Cruz Biotechnology) antibodies for 30 min at 4 °C. Cells were then washed (3×) in FACS buffer and FITC fluorescence was measured on a FACSCalibur flow cytometer (Becton Dickinson). Data analysis was performed using FlowJo software.

### In vivo studies

Donor mice bearing MIA PaCa-2 and PANC-1 tumours were placed under deep isoflurane anaesthesia and euthanised by opening the pleural cavity. Tumours were harvested rapidly and immersed in sterile, ice-cold tissue culture medium. Fragments of these tumours (2 × 2 × 2 mm) were then implanted under isoflurane anaesthesia subcutaneously in the abdominal wall of male CB17 severe combined immunodeficient (SCID) mice (strain C.B Igh-1^b^ lcrTac-Prkdc^SCID^, which were obtained from a licensed breeding colony of the Roswell Park Comprehensive Cancer Center). Topical Marcaine was applied to the skin as an analgesic and the wound was closed with a single surgical staple. The staple was removed when the wound healed after approximately 10 days. When tumour volumes averaged 300–500 mm^3^, mice were randomised into study groups having comparable mean starting tumour volumes and group standard deviations, using Microsoft Excel sorting. Mice were then treated *via* intravenous injection with volumes of ≤150 µL on days 0 and 8 of the study, in the morning. Studies included five arms in total, consisting of three experimental groups (receiving doses of CTX-MMAE at 5, 1 or 0.1 mg/kg in saline) and two control groups (receiving saline or CTX at 5 mg/kg in saline). Tumour volume was calculated as: (length × width × depth)/2. All procedures were performed in an assigned space of the Roswell Park Division of Laboratory Animal Shared Resources (LASR) under sterile conditions inside a class II laminar flow Bioguard hood.

### Quantitative modelling of CTX-MMAE

A population K-PD model was developed to describe the growth of MIA PaCa-2 and PANC-1 tumours in mice after CTX-MMAE treatment. In the absence of high-quality PK data for the antibody and its MMAE cargo in plasma, organs and tumour, which was not feasible to obtain in these studies, the PK was estimated based on a virtual one-compartment model that represents the biophase interface between plasma and tumour. The prediction of the parameters in the virtual PK model was solely dependent on the PD data,^16^ thus resting on the key, reasonable assumption that the PK of CTX-MMAE in the CB17 SCID mice was identical, for both tumours, up to the point at which the ADC was delivered to the tumour. The dynamics of tumour growth were characterised by a logistic function, which assumed that tumour volume would reach a plateau after continued growth. In CTX-MMAE-treated groups, the tumour killing effect was driven by the quantity of CTX-MMAE in the virtual PK compartment, the plasma/tumour biophase interface. The equations for the K-PD model are as follows:

1 $$\frac{{{\it{dX}}}}{{{\it{dt}}}} = - {\it{k}}_{{\it{{\mathrm{el}}}}} \cdot {\it{X}}$$

2 $$\frac{{{\it{d{\mathrm\it{TV}}}}}}{{{\it{dt}}}} = {\it{k}}_{\mathrm{g}} \cdot {\it{TV}} \cdot \left( \it{1 - \frac{{{\it{{\mathrm\it{TV}}}}}}{{{\it{{\mathrm\it{TV}}}}_{{\it{{\mathrm{max}}}}}}}} \right) - {\it{k}}_{{\it{{\mathrm{kill}}}}} \cdot {\it{X}} \cdot {\it{{\mathrm\it{TV}}}}$$

where *X* is the amount of CTX-MMAE in the virtual PK compartment; *TV* is the tumour volume at time *t*; *k*_el_ is the elimination rate constant of CTX-MMAE from the virtual PK compartment; *k*_g_ and *k*_kill_ are the tumour growth and killing rate constants, and *TV*_max_ is the maximal tumour volume. Data for tumour volume progression of MIA PaCa-2 and PANC-1 tumours were simultaneously co-modelled, with *k*_el_, *TV*_max_ and tumour volume at baseline (*TV*_0_) shared by the two tumours (the common population), leaving just two tumour-specific, fitted terms for MIA PaCa-2 *vs.* PANC-1, *k*_g_ and *k*_kill_. Between-subject variability, which followed a log-normal distribution, was included for all parameters except *TV*_max_, which was fixed to 4000 mm^3^ in the final model to avoid unidentifiability. A parameter sensitivity analysis was undertaken to evaluate the impact of each parameter on the model-predicted tumour growth. The dynamics of tumour growth in the two tumour models were also simulated for different dose regimens of CTX-MMAE that resulted in equivalent cumulative doses. All modelling and simulations were conducted using MONOLIX2018R2 (Lixoft, Antony, France) and Berkeley Madonna 9.1.14 (UC at Berkeley, Berkeley, CA).

### Data analysis

Data plotting and statistical analysis were performed on GraphPad Prism version 7 (San Diego, CA) and R version 3.5.1 (Rstudio Inc., Boston, MA). Data presented as mean ± SEM.

## Results

### Construction and characterisation of CTX-MMAE

**Fig. 1:**
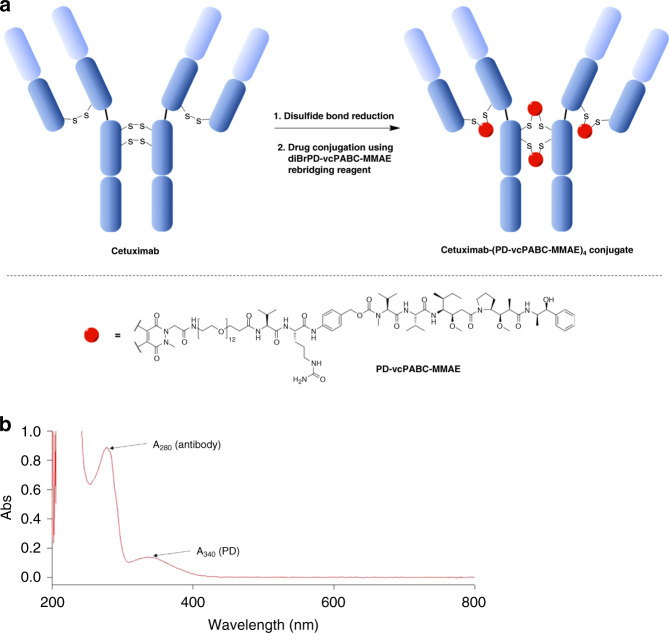
Synthesis and characterisation of CTX-MMAE. **a** Bioconjugation of CTX using a diBrPD-PEG12-valine-citrulline-PABC-MMAE re-bridging reagent to achieve the desired CTX-MMAE ADC (major product drawn, hinge intra-chain pyridazinedione-reconnectivity also observed). **b** UV-VIS absorbance spectrum of CTX-MMAE.

To enable functional re-bridging of the four native disulfides of CTX, a diBrPD-PEG12-valine-citrulline-PABC-MMAE molecule was initially synthesised^15^ (Fig. 1a). We chose MMAE as a suitable ADC payload in view of its successful application in various ADCs including FDA-approved Adcetris®. In order for MMAE to exert cytotoxic effects, it must be released from the antibody upon endocytosis. Thus, a common cleavable linker design was employed for this purpose: a cathepsin B labile valine–citrulline linker with a self-immolating PABC spacer. Conjugation of MMAE to CTX through this linker was achieved with excellent efficiency, affording a DAR of 3.9, based upon UV-VIS (Fig. 1b), HIC, and SDS-PAGE analysis (Supplementary Fig. 1), and an impressive 83% yield.

### In vitro cytotoxicity of CTX-MMAE against PaCa cell lines having differential EGFR expression

**Fig. 2:**
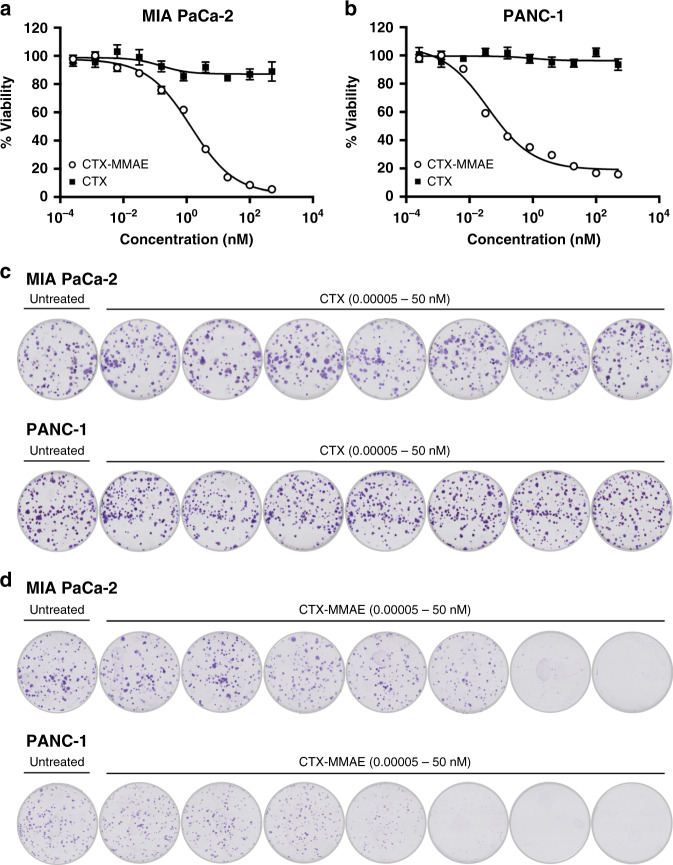
Concentration-dependent reduction in PaCa cell viability by CTX-MMAE. Viability of MIA PaCa-2 (**a**) and PANC-1 (**b**) cells following treatment with a 5-fold dilution series of CTX or CTX-MMAE ranging from 0.000256 to 500 nM for 96 h. Colony formation by MIA PaCa-2 and PANC-1 cells following treatment with a 10-fold dilution series of CTX (**c**) or CTX-MMAE (**d**) ranging from 0.00005 to 50 nM. Representative images shown.

Having successfully armed CTX with an auristatin warhead, the next series of studies evaluated the cytotoxicity of the conjugate in vitro. The KRAS mutant MIA PaCa-2 and PANC-1 PaCa cell lines were selected for these experiments because of their differential expression of EGFR. Western blot analysis (Supplementary Fig. 2) was consistent with published literature^17–19^ demonstrating that MIA PaCa-2 cells show low EGFR protein expression, whereas EGFR levels are comparatively higher on the PANC-1 line. Differential expression is potentially the result of transcriptional regulation, given that 4-fold higher EGFR mRNA expression is observed in PANC-1 cells based upon transcriptional analysis (https://depmap.org/portal/). Treatment of both lines with CTX-MMAE revealed a concentration-dependent reduction in cell viability after 96 h of exposure, with half-maximal inhibitory concentrations (IC_50_) of 1377 pM for MIA PaCa-2 and 39 pM for PANC-1 (Fig. 2a, b). In contrast, treatment with CTX alone showed a negligible effect on MIA PaCa-2 and PANC-1 viability, consistent with the known resistance of KRAS mutant tumours to this antibody.^20^ Similar trends were also noted following cell survival analysis by clonogenic assay, in which treatment with CTX-MMAE led to a concentration-dependent reduction in the colony-forming ability of both MIA PaCa-2 and PANC-1 cells (Fig. 2c, d).

### EGFR-dependent cytotoxicity of CTX-MMAE

**Fig. 3:**
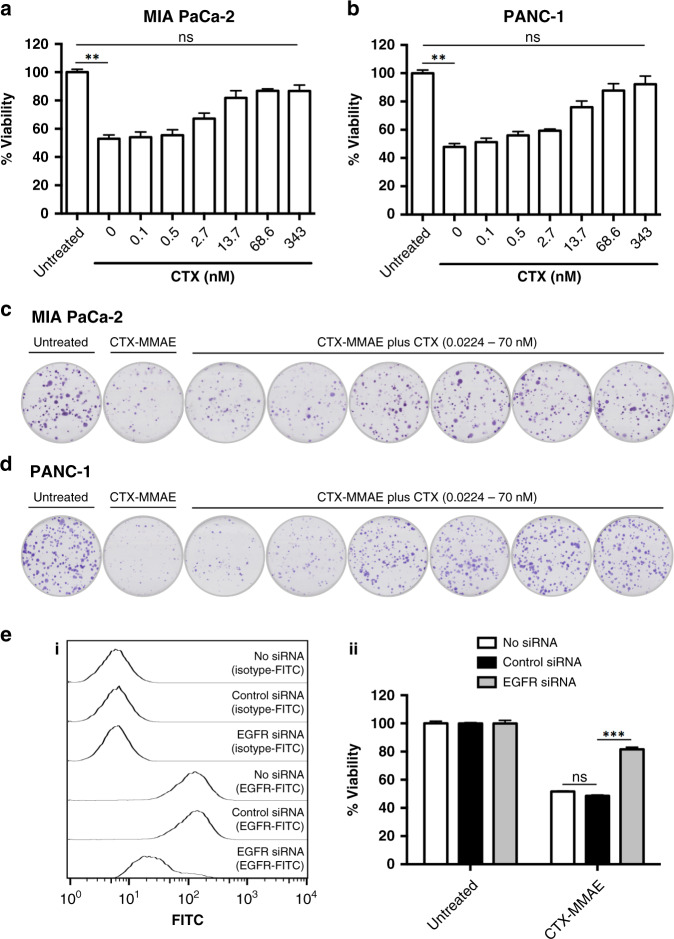
EGFR targeting specificity of CTX-MMAE. Viability of MIA PaCa-2 (**a**) and PANC-1 (**b**) cells following treatment with 5 nM CTX-MMAE ± a 5-fold dilution series of competing CTX ranging from 0.1 to 343 nM for 96 h. Statistical significance was determined by Kruskal–Wallis test with Dunn’s post hoc analysis (**p ≤ 0.01, ns *p* > 0.05). Colony formation by MIA PaCa-2 (**c**) and PANC-1 (**d**) cells following treatment with 0.5 nM CTX-MMAE ± a 5-fold dilution series of competing CTX ranging from 0.0224 to 70 nM. Representative images shown. **e** PANC-1 cells were subjected to a mock transfection (no siRNA) or transfected with control siRNA or EGFR siRNA. (i) Flow cytometric analysis of PANC-1 cells stained with a FITC-labelled EGFR antibody or isotype control antibody at 72 h post transfection. Representative histograms shown for each of the annotated samples. (ii) Viability of transfected PANC-1 cells following treatment with 0.5 nM CTX-MMAE for 72 h. Statistical significance was determined by Kruskal–Wallis test with Dunn’s post hoc analysis (***p ≤ 0.001, ns *p* > 0.05).

Several approaches were next employed to confirm that these cytotoxic effects were mediated *via* EGFR. Competition studies were initially performed, in which MIA PaCa-2 and PANC-1 cultures were simultaneously exposed to CTX-MMAE and various concentrations of CTX. Endpoint MTT analysis demonstrated that CTX inhibited the cytotoxicity of CTX-MMAE in a concentration-dependent manner, indicating that the cytotoxicity of CTX-MMAE is dependent on the presence of cell-surface EGFR (Fig. 3a, b). These findings were corroborated by clonogenic assays, in which colony formation was progressively restored to similar levels as the untreated controls upon co-treatment with CTX-MMAE and increasing concentrations of competing CTX (Fig. 3c, d). To further verify these findings, we employed RNA interference as an independent technique and confirmed efficient knockdown of cell-surface EGFR (Fig. 3ei). Whereas treatment with CTX-MMAE induced potent cell death in PANC-1 cultures that were subjected to a mock or a control siRNA transfection, these effects were significantly alleviated upon knockdown of EGFR (Fig. 3eii). Collectively, these data provide robust confirmation of the EGFR targeting specificity of CTX-MMAE.

### In vivo efficacy of CTX-MMAE in xenograft models of PaCa

**Fig. 4:**
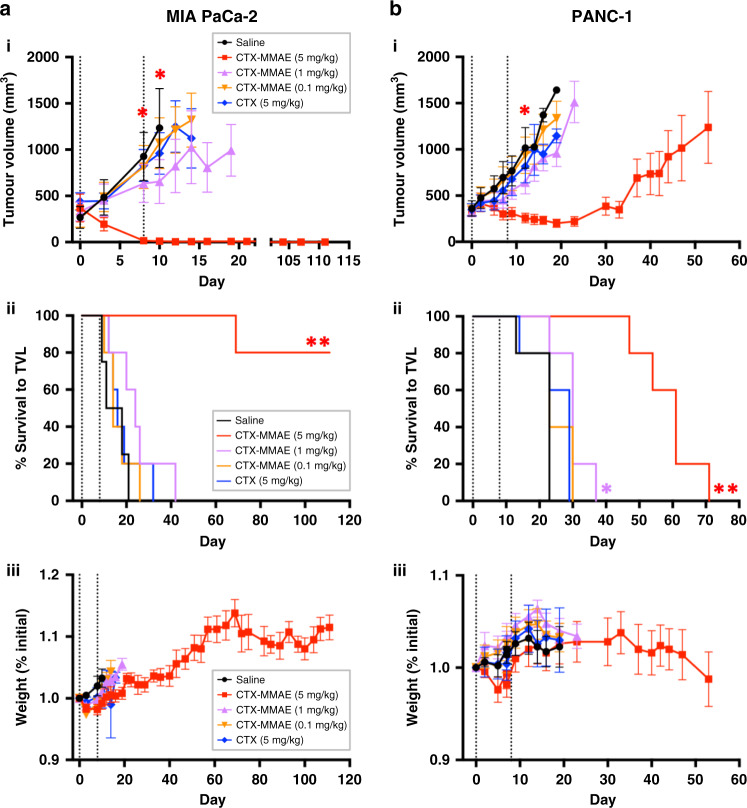
Therapeutic efficacy of CTX-MMAE in xenograft models of PaCa. CB17 SCID mice bearing subcutaneous **a** MIA PaCa-2 (*n* = 4–5 per group) and **b** PANC-1 (*n* = 5 per group) xenografts were intravenously injected with saline, CTX (5 mg/kg) or CTX-MMAE (0.1, 1 and 5 mg/kg) on day 0 and 8 of the study (indicated by vertical dashed lines). (i) Mean tumour volume was plotted until the second of two animals in each treatment group reached the maximum TVL of 2000 mm^3^. Statistical significance between the CTX-MMAE (5 mg/kg) and saline treatment groups was determined by one-way analysis of variance (ANOVA) with Bonferroni post hoc analysis (*p ≤ 0.05). (ii) Kaplan–Meier survival analysis for each study arm in (i), based on time of tumour progression to a TVL of 2000 mm^3^. Statistical significance between the CTX-MMAE and saline treatment groups was determined by log-rank test (*p ≤ 0.05, **p ≤ 0.01). (iii) Body weight analysis for each study arm in (i). Measurements were plotted until the second of two animals in each treatment group reached the maximum TVL of 2000 mm^3^.

The therapeutic activity of CTX-MMAE was next evaluated in vivo in SCID mice bearing subcutaneous MIA PaCa-2 or PANC-1 xenografts. When tumours reached a starting volume of ~300–500 mm^3^, mice were dosed intravenously with CTX-MMAE, CTX, or saline on days 0 and 8 of the study. In mice implanted with MIA PaCa-2 xenografts, which have a lower EGFR density than PANC-1, and a 35-fold higher IC_50_ for CTX-MMAE, treatment with CTX-MMAE at 0.1, 1 or 5 mg/kg led to dose-dependent inhibition of tumour growth. Within 8 days of dosing, the highest CTX-MMAE dose group (5 mg/kg) was statistically smaller than controls (*p* ≤ 0.05). By day 10 following initiation of treatment, at which time the control group reached a tumour threshold volume limit (TVL) of 2000 mm^3^, mean tumour volumes were reduced by 13% (0.1 mg/kg), 47% (1 mg/kg) and 99% (5 mg/kg) relative to the control arm (Fig. 4ai). At the highest dosing level of CTX-MMAE (5 mg/kg), 4/5 mice experienced complete and durable tumour regressions, with no recurrences observed before the 111-day study endpoint. In contrast, a 5 mg/kg dose of the naked antibody CTX did not alter tumour growth compared to the control arm. Whereas median time to the TVL was 14.5 days for the saline control group, 4/5 mice treated with 5 mg/kg of the conjugate survived without progression to the 111-day study endpoint, and a median survival to TVL could not be calculated (Fig. 4aii, Tables 1 and 2). Neither body weights (Fig. 4aiii) nor body condition were adversely affected, confirming that CTX-MMAE was well-tolerated by all mice.

**Table 1:** Median survival time in all treatment groups.

	Median survival to TVL (days)	
Treatment groups	MIA PaCa-2	PANC-1
Saline	14.5	23
CTX-MMAE (0.1 mg/kg)	14	23
CTX-MMAE (1 mg/kg)	24	30
CTX-MMAE (5 mg/kg)	NA	61
CTX (5 mg/kg)	16	29

**Table 2:** Statistical analysis of Kaplan–Meier curves.

		*p*-value for log-rank test of Kaplan–Meier curves	
Treatment group comparisons		MIA PaCa-2	PANC-1
Saline	CTX-MMAE (0.1 mg/kg)	0.65	0.28
Saline	CTX-MMAE (1 mg/kg)	0.059	**0.014**
Saline	CTX-MMAE (5 mg/kg)	**0.0067**	**0.0035**
CTX-MMAE (0.1 mg/kg)	CTX-MMAE (1 mg/kg)	0.22	0.14
CTX-MMAE (0.1 mg/kg)	CTX-MMAE (5 mg/kg)	**0.0046**	**0.0026**
CTX-MMAE (1 mg/kg)	CTX-MMAE (5 mg/kg)	**0.0049**	**0.002**

The *p-*values that are statistically significant at *p* ≤ 0.05 are highlighted in bold to emphasise differences between the two treatment arms that are significant.

The same treatment regimen was also tested in PANC-1 xenografts, which have a higher EGFR density, and greater in vitro sensitivity to CTX-MMAE, than MIA PaCa-2. Whereas 5 mg/kg CTX-MMAE led to complete regression in the MIA PaCa-2 model, PANC-1 xenografts showed a more modest initial reduction in tumour volume during treatment. By day 12 after initiation of dosing, the 5 mg/kg CTX-MMAE dose group was statistically smaller than controls (*p* ≤ 0.05). However, regrowth was observed ~25 days after the completion of treatment (Fig. 4bi). Kaplan–Meier analysis revealed that the highest dose of CTX-MMAE, 5 mg/kg, almost tripled the median survival time to the TVL relative to saline-treated controls (*p* < 0.005), whereas the 1 mg/kg CTX-MMAE group was also statistically different from controls (*p* < 0.05) (Fig. 4bii, Tables 1 and 2). Body weights remained consistent throughout the study, with no indications of toxicity (Fig. 4biii). Taken together, these studies demonstrate the marked efficacy and apparent tolerability of CTX-MMAE in models of PaCa that differ in expression of the target receptor EGFR.

### Quantitative analysis to investigate the dose-efficacy relationship of CTX-MMAE

**Fig. 5:**
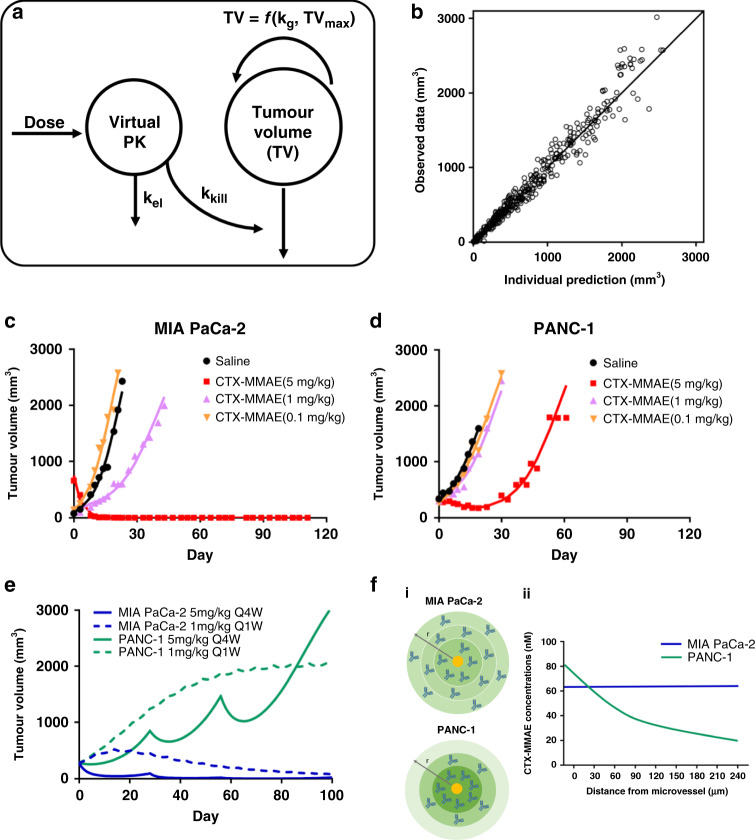
Population K-PD modelling of CTX-MMAE in xenograft models of PaCa. **a** Schematic of the CTX-MMAE K-PD model. The circles represent the virtual PK compartment and the tumour volume compartment; *k*_el_ is the CTX-MMAE elimination rate constant in the virtual PK compartment, and *k*_g_ and *k*_kill_ are the tumour growth and killing rate constants. The amount of CTX-MMAE in the virtual PK compartment is the driver for the tumour killing effect. **b** Diagnostic plot for model fittings: measured tumour volume *vs.* individual prediction. The uniform distribution of scatter points along the diagonal line indicates the goodness-of-fit of the model. **c**, **d** Representative individual model fittings of tumour volume progression in the MIA PaCa-2 and PANC-1 treatment groups. All individual tumour volume data for the different treatment groups for both tumours were fitted simultaneously with the final model, and the figures show the model fitting of tumour dynamics for representative mice from each treatment group. **e** Simulations of tumour volume progression with different dose regimens for the two tumour models. Tumour volumes were simulated over a period of 100 days for administration of 1 mg/kg Q1W CTX-MMAE for fifteen cycles and 5 mg/kg Q4W CTX-MMAE for three cycles in MIA PaCa-2 and PANC-1 tumours. **f** (i) Schematic illustration of hypothetical CTX-MMAE distribution in MIA PaCa-2 and PANC-1 tumours viewed as a tumour cross-section. The yellow circle represents a microvessel. The darkness of the green circle represents the relative concentrations in different regions of the tumour. With relative low expression of EGFR in the MIA PaCa-2 tumour, the receptors that are proximal to the microvessel are saturated readily, allowing more free CTX-MMAE to distribute into the distal tumour regions homogenously. With high expression of EGFR in the PANC-1 tumour, the capacity of cells proximal to the microvessel to bind CTX-MMAE is greater, and because of this ‘binding site barrier’, less CTX-MMAE is available to distribute into the distal regions of the tumour. (ii) Hypothetical CTX-MMAE concentrations as a function of distance from the microvessel in MIA PaCa-2 and PANC-1 tumours based on preliminary CTX tumour PK data.

Experimental data from the in vivo studies was used to develop a K-PD model to analyse the tumour response dynamics of the two pancreatic tumours to differing CTX-MMAE doses (Fig. 5a). K-PD models represent a comparatively new paradigm to leverage the response *vs.* time profiles from multiple dose levels and for multiple tumours by hypothesising the existence of a common hypothetical driver,^21^ particularly in cases such as this, where obtaining the necessary high-quality PK data for the ADC and its linked drug can prove challenging. All efficacy data for both tumours were modelled simultaneously, and the final K-PD model captured tumour volume progression well for the two human xenografts, as seen from the diagnostic plot of observed *vs.* predicted tumour volume (Fig. 5b). The data are distributed evenly along the diagonal observed *vs*. predicted line, demonstrating reasonable model fittings. Figure 5c, d show the model fittings of tumour volume progression for representative individual mice from each treatment arm for both tumour models. The population parameters were estimated with good precision, although inter-individual variability (IIV) was estimated with a relatively large uncertainty because of the comparatively small number of mice in each group (Table 3). The estimated tumour growth rate *k*_g_ for the MIA PaCa-2 tumour was slightly larger than that of PANC-1, consistent with the shorter doubling time of MIA PaCa-2 cells observed in vitro and in vivo.

**Table 3:** Population K-PD model parameter estimates.

Parameters (unit)	Description	MIA PaCa-2		PANC-1	
		Mean (%RSE)	%IIV (%RSE)	Mean (%RSE)	%IIV (%RSE)
*k*_el_ (day^−1^)	Elimination rate constant for virtual PK	0.0776 (34.2)	104 (30.6)	Same as MIA PaCa-2	
*k*_g_ (day^−1^)	Exponential tumour growth rate constant	0.152 (9.25)	26.4 (28)	0.111 (3.13)	5.18 (50.9)
*TV*_0_ (mm^3^)	Tumour volume at baseline	281 (10.7)	65.2 (11.9)	Same as MIA PaCa-2	
*TV*_max_ (mm^3^)	Maximal tumour volume	4000 (fixed)	–	Same as MIA PaCa-2	
*k*_kill_ (mg^−1^ • day^−1^)	Killing rate constant	3.70 (22)	33.2 (57.9)	1.18 (9.30)	15.2 (52.9)

*IIV* inter-individual variability, *RSE* relative standard error.

Because of the protocol TVL of 2000 mm^3^, no tumour volume progression data could be obtained for the control group beyond that volume limit, and as a result, the maximal unperturbed tumour volume (*TV*_max_) would not be estimated well by the model. Therefore, it was fixed to 4000 mm^3^. Based on model fittings in which the fixed values of *TV*_max_ were varied, this virtual maximal volume showed little impact on overall conclusions (Table 3). Notably, the model-estimated *k*_kill_ for MIA PaCa-2 tumours was two-fold higher than that of PANC-1, consistent with the observation that CTX-MMAE was more efficacious in the MIA PaCa-2 xenograft tumour, despite the higher EGFR density on PANC-1. Parameter sensitivity analysis indicated that tumour volume progression is most sensitive to *k*_g_ and *k*_kill_, and to *k*_el_, which is the elimination rate constant for the virtual PK model component (Supplementary Fig 3).

To investigate which factors exert greatest impact upon treatment efficacy, as well as explore how dose and dosing frequency might affect outcomes, the dynamics of tumour growth were simulated under dosing regimens that included lower doses given more frequently and higher doses given less frequently, yet still achieving the same cumulative dose. Figure 5e shows model simulations of tumour growth with cumulative doses of 15 mg/kg CTX-MMAE given as 1 mg/kg once weekly (Q1W) for fifteen cycles or as 5 mg/kg given once per month (Q4W) for three cycles. For the MIA PaCa-2 tumour, predictions with the model suggest that both a low dose of CTX-MMAE administered weekly for a longer period and a higher dose administered less frequently could suppress tumour growth in a sustained manner for up to at least 100 days, although the higher dose was predicted to suppress tumour volume more rapidly. Consistent with experimental findings, simulations with the model also suggested that the higher-EGFR PANC-1 tumour would continue to progress under either regimen.

## Discussion

ADC development has faced numerous challenges that have significantly hindered progress within the field until recently. In particular, traditional methods for coupling cytotoxic warheads to antibodies are typically based on random and uncontrolled conjugation to lysine or cysteine residues, leading to heterogeneous conjugates with a distribution of DARs and suboptimal pharmacological properties. Here, we report a significant advance towards the development of next-generation homogeneous ADCs, based on reduction of the four inter-chain disulfide bonds of CTX and their subsequent re-bridging by thiol-reactive diBrPD-based linkers appended with MMAE. This approach affords exceptional control over the positioning and number of MMAE molecules coupled to CTX, resulting in the generation of highly refined conjugates with a DAR of 3.9 and potent therapeutic activity in PaCa models. The exciting potential of this re-bridging technology is also supported by the work of Li et al. who employed a similar strategy to construct an ADC composed of an in-house EGFR antibody and a MMAE payload, for therapy of KRAS wild-type PaCa xenografts.^22^ Here, we contribute further significant advances to the field through the demonstration of CTX-MMAE efficacy in KRAS mutant models that reflect the high frequency of these mutations in PaCa patients, together with the inclusion of a predictive modelling tool to guide the successful application of our ADC.

In addition to disulfide re-bridging, other site-specific bioconjugation strategies can improve ADC homogeneity, such as incorporation of additional cysteines or unnatural amino acids, enzyme-assisted ligation, and glycan modification.^23–26^ However, these approaches necessitate expensive and/or arduous protein engineering, may potentially invoke immunogenic effects, and are not readily transferable to all antibody platforms without individualised optimisation. Given that the strategy employed here is based on re-bridging of native disulfide bonds located distal to the paratopes, it may be universally applicable to all antibodies, with minimal perturbation of their structural integrity, stability and binding activity. These attributes represent a distinct advantage over various other site-specific coupling approaches and are likely to expedite the ADC development process from both manufacturing and regulatory perspectives. Nonetheless, we acknowledge current limitations of our disulfide re-bridging approach, in which these proof-of-concept conjugations were performed with a 10-fold excess of the diBrPD-PEG12-valine-citrulline-PABC-MMAE drug-linker. Optimisation of the synthetic route is warranted going forward, given the demonstration that CTX-MMAE is highly active in vivo against multiple PaCa xenograft tumours.

Our findings have important implications for CTX-based therapy in PaCa. Overexpression of EGFR has been reported in >90% of pancreatic tumours and has also been shown to correlate with poorer prognosis.^27^ Despite these observations providing a clear rationale for the use of CTX in PaCa, it has so far failed to impart a meaningful clinical benefit in this tumour setting when combined with other frontline agents,^28–30^ most likely because of the concomitant high frequency of KRAS mutations in this disease. In notable consistency with clinical observations, both of the EGFR-positive pancreatic cell lines investigated here were highly resistant to CTX treatment. However, we demonstrate that CTX, once armed with an ultrapotent MMAE warhead, can mediate profound antitumour effects *via* an EGFR-dependent mechanism. These findings identify a new therapeutic opportunity for CTX and potentially other EGFR-targeted antibodies in PaCa, whereby it may be repurposed as a targeted drug delivery platform. This strategy may also find application in other EGFR-positive tumours in which CTX has been ineffective, such as in KRAS mutant colorectal cancers.^20,31^

As in clinical studies, obtaining accurate PK data for both the antibody and the ultrapotent ADC warhead is extremely challenging. Development of a K-PD model circumvents this challenge by driving CTX-MMAE tumour PD with a virtual PK compartment that employs the reasonable assumption that the PK of the ADC up to the point of the tumour biophase interface is equivalent in both tumours. The final K-PD model captured well the PD responses of multiple dose levels and treatments in two xenografts, with just two parameters that were tumour-specific, and provided interesting insights into the differences observed in CTX-MMAE efficacy in MIA PaCa-2 *vs*. PANC-1 xenografts. The inter-subject variability in tumour volume progression within treatment groups, and the protocol requirement for withdrawal of mice from the study when the tumour reached a limit of 2000 mm^3^, made a comparison of average tumour volumes across treatment groups at a single time point an inferior approach to analyse in vivo efficacy. In addition, the differing growth rates of the two tumour models would complicate a comparison of CTX-MMAE efficacy between them. The K-PD model, which integrated virtual PK, tumour-specific growth rates, and tumour killing effects of CTX-MMAE, allowed quantitative prediction of the dose-efficacy relationship and provided a parameter estimating the relative in vivo potency of CTX-MMAE (*k*_kill_) for each tumour model. Utilisation of a population modelling approach to account for the impact of inter-subject variabilities in tumour growth and response enabled good prediction of the central tendency of the model parameters. The analysis demonstrated that CTX-MMAE showed higher in vivo potency in the MIA PaCa-2 tumour compared to PANC-1 (*k*_kill_: 3.70 mg^−1^ day^−1^ *vs.* 1.18 mg^−1^ day^−1^) (Table 3), which runs counter to the in vitro finding that CTX-MMAE was more potent on PANC-1 tumour cells, and the fact that PANC-1 cells have higher EGFR expression; higher target receptor expression would be expected to mediate greater internalisation of the cytotoxic payload. In vitro, all tumour cells are directly accessible to CTX-MMAE in the medium, and the higher observed potency in the PANC-1 model can be attributed to more receptor binding and internalisation of CTX-MMAE. In contrast, the determinants of ADC activity in vivo are complex. They include tumour vascularity and perfusion, rate and magnitude of tumour deposition, intra-tumour ADC distribution, which is mediated by diffusion or convection, target receptor density, rate of ADC-receptor complex internalisation, intracellular release rate of the MMAE warhead, and bystander effects of MMAE. Preliminary experiments, employing randomly-labelled fluorescent CTX as a surrogate for CTX-MMAE, suggest that initial CTX uptake is more rapid in MIA PaCa-2 tumours, but over 96 h, CTX tumour deposition is equivalent in the two tumours.

A hypothesis to explain the lower potency of CTX-MMAE on the PANC-1 tumour in vivo, despite its higher EGFR expression and greater in vitro sensitivity, is that the greater abundance of high-affinity receptors proximal to afferent microvessels would deplete the inward flux of ADC, constituting a ‘binding site barrier’ (Fig. 5fi) that would reduce tumour penetration of CTX-MMAE.^32^ Although the PANC-1 tumour cells near microvessels would be killed efficiently by CTX-MMAE, those at a greater distance from microvessels would experience lower ADC exposure, potentially escaping killing (Fig. 5fii). By this reasoning, MIA PaCa-2 tumours, having a lower abundance of EGFR, would not deplete the inward flux of CTX-MMAE to as great an extent as PANC-1; therefore, greater numbers of cells would be eradicated, resulting in greater overall efficacy. Simulations with the K-PD model, shown in Fig. 5e, predict that even with lower doses of CTX-MMAE given more frequently, or higher doses given less frequently, PANC-1 tumour progression would not be controlled, despite greater intensity of killing by the higher dose, whereas MIA PaCa-2 tumour growth suppression would be durable long-term with either dosing regimen. These hypotheses bear future experimental testing and analysis with mechanistic PK-PD models that are able to estimate the influence of the multiple factors affecting tumour cell killing by CTX-MMAE, including receptor density and tumour distribution of the ADC. Moreover, another interesting factor for future investigation will be the impact of tumour volume on ADC efficacy. Examination of whether the ‘binding site barrier’ becomes less prominent in smaller tumours will be of particular interest, which could have important positive implications for the treatment of advanced PaCa where micrometastases have established.

Our experimental findings lead to the obvious conclusion that EGFR expression would be an important biomarker for selection of patients most likely to respond to CTX-MMAE therapy. However, given the initially counter-intuitive observation of lower efficacy of the ADC on the higher EGFR-expressing PANC-1 tumour, density of expression alone may not correlate with improved ADC efficacy. A further factor that may impact the biomarker status of EGFR is the extent of bystander cytotoxicity elicited by CTX-MMAE, which has been documented for other MMAE-containing ADCs such as clinically approved Adcetris®.^33^ These effects are facilitated by the low molecular weight and lipophilicity of MMAE, which allow drug released from the antibody linker to diffuse readily from EGFR-positive target cells and subsequently permeate neighbouring cells regardless of their target antigen expression. Evaluation of CTX-MMAE bystander killing will be an important objective going forward, given its clinical importance in tumours of mixed- or varying target receptor status.

Also of key importance moving forward will be to investigate activity of CTX-MMAE on a larger panel of PaCa models such as patient-derived xenograft (PDX) models that have clinically-relevant, varying levels of EGFR expression, and recapitulate the complex histology and characteristics of clinical PaCa isolates. Combined with appropriate, mechanistic, next-generation PK-PD models, these studies will facilitate in-depth exploration of the role of the ‘binding site barrier’ in CTX-MMAE efficacy and how it could potentially be overcome. One recent strategy employed co-administration of unladen antibodies to pre-block tumour receptors proximal to the vasculature partially, so as to enhance deeper tumour penetration of ADCs.^34^ Other alternatives worthy of investigation include ‘tumour priming’ strategies that compromise the tumour/blood permeability barrier, as well as the convection/diffusion barriers constituted by the tumour stroma, to increase the volume of tumour that is accessible to plasma-borne ADCs.^35–39^ In particular, targeting the vasculature through co-administration of agents known to ‘normalise’ vessel perfusion and functionality could potentially enhance tumoural delivery of CTX-MMAE.

In summary, we have demonstrated the ultrapotent and sustained antitumour effects of a next-generation CTX-MMAE ADC in PaCa models, constructed using a state-of-the-art linker technology that enables highly controlled, site-specific coupling of drug molecules to antibodies. Despite disappointing clinical outcomes in PaCa patients treated with the parental CTX antibody, in spite of the nearly ubiquitous overexpression of EGFR in their cancers, our findings suggest that CTX may be repurposed as a highly effective, targeted delivery platform to courier cytotoxic drugs such as MMAE to PaCa cells. This strategy therefore has the potential to exploit EGFR overexpression in tumours that are otherwise protected from anti-EGFR treatment strategies by reason of their KRAS mutations.

## Supplementary information

Supplementary material

## Data Availability

All data and material requests should be directed to C.J.S. (c.scott@qub.ac.uk), V.C. (v.chudasama@ucl.ac.uk) or R.M.S. (rms@buffalo.edu).
