# Supplementary material for: Controlled coupling of an ultrapotent auristatin warhead to cetuximab yields a next-generation antibody-drug conjugate for EGFR-targeted therapy of KRAS mutant pancreatic cancer
Source: Br J Cancer. 2020 Sep 11;123(10):1502–12. doi: 10.1038/s41416-020-01046-6 (PMC7653048; doi:10.1038/s41416-020-01046-6)
Supplement: Supplementary file 1 — Supplementary material [file 41416_2020_1046_MOESM1_ESM.docx]

**Supplementary material**

**Supplementary Figure 1. Characterisation of CTX-MMAE.** (A) SDS-PAGE analysis (boiling in some SDS (a denaturing agent) to show what the precise nature is for analysis purposes; the Fc region antibody chains are in fact highly templated and in the absence of boiling in SDS are inseparable independent of disulfide hinge connectivity). (B) HIC analysis of CTX-MMAE indicated an average DAR of 3.9, with a DAR of 4 being the major product (76%).

**Supplementary Figure 2. EGFR expression on PaCa cell lines.** Western blot analysis of EGFR protein expression in whole cell lysates harvested from MIA PaCa-2 and PANC-1 cultures. Tubulin expression was also probed as a loading control. Representative immunoblots shown.

**Supplementary Figure 3**. **Sensitivity of tumour volume progression to key parameter values.** The sensitivity of tumour growth simulations by the K-PD model of Figure 5 was tested by individually altering key parameters in the range of their final estimated values: (A) tumour volume at baseline (TV_0_); (B) maximal tumour volume (TV_max_); (C) elimination rate constant from virtual PK model component (k_el_); (D) tumour growth rate constant (k_g_); (E) killing rate constant (k_kill_) on tumour growth. The K-PD model was run with each of the individual parameters changed by ½X and 2X of their final values (TV_0_=281 mm^3^, TV_max_=4000 mm^3^, k_el_=0.0776 day^-1^, k_g_=0.132 day^-1^ and k_kill_=2.44 mg^-1^day^-1^).

**Animal Research: Reporting of *In Vivo* Experiments (ARRIVE)**

One of the key objectives of this work was to test the therapeutic activity of CTX-MMAE in a tumour model that closely mimics the human setting. Murine xenografts of human cancers are widely considered as gold standard models for such purpose, given that mice share many common traits with humans (e.g. significant overlap of genes and signalling pathways involved in human cancers), and were therefore employed in this work. All work with animals was approved in advance by the Institutional Animal Care and Use Committee (IACUC) of Roswell Park Comprehensive Cancer Center (Buffalo, NY), and the approval was reviewed and certified by the IACUC of the University at Buffalo, State University of New York. Animals were housed in a pathogen-free environment in individually ventilated cages, which were held on racks fitted with automatic watering ports. Water was purified by reverse osmosis, using a computer-controlled, self-flushing water distribution system. Cage bedding consisted of crushed corn-cob and animals were fed *ad libitum* with autoclaved 18% protein standard rodent diet. Animals were maintained under a 12 hr light : 12 hr dark cycle at a constant temperature of 70 ± 2 ^o^F and relative humidity between 30 – 70%. A total of 24 – 25 treatment-naïve animals were used for each xenograft study (aged 6 – 8 weeks and weighing 18 – 25 g), which were split into groups of 4 – 5 that comprised an experimental unit. These animal numbers ensured that studies were sufficiently powered to allow detection of a statistically significant difference between groups. Doses and routes of administration were determined based on published literature in which ADCs had similarly been administered to animals bearing subcutaneous tumours. The approved IACUC protocol prescribed anaesthesia and analgesia during surgical procedures, as well as methods of euthanasia. Animals were treated and assessed according to previously random tail markings from 1 – 5 (or 1 – 4) for each group. Tumour volume and body weight were measured 2 – 3 times weekly by study personnel, along with overall assessment of animal body condition and behavioural endpoints in accordance with specific rating criteria. Roswell Park LASR core facility staff and veterinarians regularly performed independent assessments of tumour size, body condition, and behaviour. The IACUC-approved protocol required that animals be removed from the study and euthanised if tumour volume reached 2000 mm^3^, if any tumour dimension reached 20 mm, if a 20% decline in body weight was experienced, or if body condition score deteriorated. Primary and secondary experimental outcomes included tumour volume, survival to the TVL of 2000 mm^3^, and body weight. Animals were euthanised by carbon dioxide exposure, followed by cervical dislocation, at study endpoint.
